# Sex Differences in Receiving Layperson Cardiopulmonary Resuscitation in Pediatric Out‐of‐Hospital Cardiac Arrest: A Nationwide Cohort Study in Japan

**DOI:** 10.1161/JAHA.118.010324

**Published:** 2018-12-27

**Authors:** Masashi Okubo, Tasuku Matsuyama, Koichiro Gibo, Sho Komukai, Junichi Izawa, Kosuke Kiyohara, Chika Nishiyama, Takeyuki Kiguchi, Clifton W. Callaway, Taku Iwami, Tetsuhisa Kitamura

**Affiliations:** ^1^ Department of Emergency Medicine University of Pittsburgh School of Medicine Pittsburgh PA; ^2^ Department of Emergency Medicine Kyoto Prefectural University of Medicine Kyoto Japan; ^3^ Department of Emergency Medicine Okinawa Prefectural Chubu Hospital Uruma Japan; ^4^ Division of Biomedical Statistics Department of Integrated Medicine Graduate School of Medicine Osaka University Suita Japan; ^5^ Department of Critical Care Medicine University of Pittsburgh School of Medicine Pittsburgh PA; ^6^ Department of Anesthesiology The Jikei University School of Medicine Tokyo Japan; ^7^ Department of Food Science Otsuma Women's University Tokyo Japan; ^8^ Department of Critical Care Nursing Kyoto University Graduate School of Human Health Science Kyoto Japan; ^9^ Kyoto University Health Service Kyoto Japan; ^10^ Division of Environmental Medicine and Population Services Department of Social and Environmental Medicine Graduate School of Medicine Osaka University Suita Japan

**Keywords:** cardiac arrest, cardiopulmonary resuscitation, gender differences, pediatric, Cardiopulmonary Arrest, Cardiopulmonary Resuscitation and Emergency Cardiac Care, Pediatrics, Women

## Abstract

**Background:**

Layperson cardiopulmonary resuscitation (CPR) is a crucial intervention for patients with out‐of‐hospital cardiac arrest (OHCA). Although a sex disparity in receiving layperson CPR (ie, female patients were less likely to receive layperson CPR) has been reported in adults, there are few data in the pediatric population, and we therefore investigated sex differences in receiving layperson CPR in pediatric patients with OHCA.

**Methods and Results:**

From the All‐Japan Utstein Registry, a prospective, nationwide, population‐based OHCA database, we included pediatric patients (≤17 years) with layperson‐witnessed OHCA from 2005 through 2015. The primary outcome was receiving layperson CPR. Patient sex was the main exposure. We fitted multivariable logistic regression models to examine associations between patient sex and receiving layperson CPR. We included a total of 4525 pediatric patients with layperson‐witnessed OHCA in this study, 1669 (36.9%) of whom were female. Female patients received layperson CPR more often than male patients (831/1669 [49.8%] versus 1336/2856 [46.8%], *P*=0.05). After adjustment for age, time of day of arrest, year, witnesses persons, and dispatcher CPR instruction, the sex difference in receiving layperson CPR was not significant (adjusted odds ratio for female subjects 1.14, 95% CI, 0.996‐1.31).

**Conclusions:**

In a pediatric population, female patients with layperson‐witnessed OHCA received layperson CPR more often than male patients. After adjustment for covariates, there was no significant association between patient sex and receiving layperson CPR.

**Clinical Perspective**

**What Is New?**

- In a nationwide cohort study of 4525 pediatric patients with layperson‐witnessed out‐of‐hospital cardiac arrest in Japan, female patients received layperson cardiopulmonary resuscitation more often than male patients, although the sex difference did not persist after adjustment for covariates.

**What Are the Clinical Implications?**

- The finding suggests the importance of public health efforts to increase provision of cardiopulmonary resuscitation for patients of both sexes.

## Introduction

Out‐of‐hospital cardiac arrest (OHCA) is a global public health problem annually affecting more than 350 000 individuals in the United States and 123 000 in Japan.^1^, ^2^ Mortality after OHCA is high despite intensive international efforts (eg, International Consensus on Cardiopulmonary Resuscitation and Emergency Cardiovascular Care Science With Treatment Recommendations) to improve patient outcomes.^3^ The pediatric population constitutes a particularly vulnerable segment of patients with OHCA, and the public health burden of pediatric OHCA remains high because of the greater number of lost years of productivity per individual.^4^

Layperson cardiopulmonary resuscitation (CPR) is a part of the “chain of survival” and plays an essential role in OHCA care.^5^, ^6^ Although resuscitation guidelines for adults and children recommend the provision of layperson CPR,^6^, ^7^, ^8^, ^9^ ≈50% of patients with emergency medical services (EMS)‐treated OHCA receive layperson CPR in the United States and Japan, suggesting an opportunity to further disseminate and implement this critical intervention.^10^, ^11^ Therefore, it is important to understand the underlying obstacles of providing layperson CPR. A few studies have showed that female adults with OHCA were less likely to receive layperson CPR than male adults.^12^, ^13^ However, any sex differences in receiving layperson CPR in a pediatric population remain unknown.

To address this knowledge gap, we analyzed the All‐Japan Utstein Registry.^11^, ^14^, ^15^, ^16^ We tested the main hypothesis that receiving layperson CPR differs between patient sexes in the pediatric population. We also tested our subhypotheses that (1) there are interactions between patient sex and age and between patient sex and witnesses on receiving layperson CPR; and (2) patient survival and functional outcomes differ between patient sexes.

## Methods

The data, analytic methods, and study materials will not be made available to other researchers for purposes of reproducing the results or replicating the procedure.

### Study Design, Setting, and Participants

We conducted a secondary analysis of the All‐Japan Utstein Registry of the Fire and Disaster Management Agency, a prospective, nationwide, population‐based registry system of OHCA that includes the entire population of Japan. The details of this registry have been previously reported elsewhere^11^, ^14^, ^15^, ^16^ This study included pediatric patients (≤17 years) with layperson‐witnessed OHCA on whom EMS attempted resuscitation from 2005 through 2015, with subsequent transport to hospitals. We defined cardiac arrest as lack of cardiac mechanical activity confirmed by lack of clinical evidence of blood circulation.^17^, ^18^, ^19^ We defined attempted resuscitation as shock delivery with external defibrillators (by laypeople or EMS personnel) or chest compression by EMS personnel.^17^, ^18^, ^19^ We excluded EMS‐witnessed arrest, unwitnessed arrest, and OHCA with unknown age, unknown witness status, unknown first documented rhythm, and unknown layperson CPR. The EMS system in Japan has been previously described elsewhere.^11^, ^14^, ^15^, ^16^ Briefly, all EMS personnel perform resuscitation in accord with the Japanese CPR guidelines, based on the International Liaison Committee on Resuscitation consensus.^20^ EMS personnel initiate resuscitation except under particular conditions (eg, decapitation, incineration, decomposition, rigor mortis, or dependent cyanosis) and are not legally permitted to terminate resuscitation in the field.^11^, ^14^, ^15^, ^16^ The majority of patients with OHCA were therefore transferred to hospitals and included in the registry. The institutional review board of Kyoto University approved the secondary analysis of the All‐Japan Utstein Registry with a waiver of informed consent.

### Data Collection and Quality Control

Data were prospectively collected using the Utstein Resuscitation Registry Templates for OHCA.^17^, ^18^, ^19^ The form included age, sex, date of cardiac arrest, etiology of cardiac arrest, witness status, characteristics of witnesses, first documented rhythm, presence and types of layperson CPR, presence of dispatcher CPR instruction, public‐access automated external defibrillator shock delivery, presence and type of prehospital advanced airway management, prehospital administration of intravenous fluids and epinephrine, and resuscitation time courses as well as patient outcome measurements: prehospital return of spontaneous circulation, 1‐month survival, and functional status at 1 month after cardiac arrest.^11^, ^14^, ^15^, ^16^ To collect 1‐month outcome data, EMS providers in charge followed up all survivors for 1 month after the arrest. Functional outcome was determined at a follow‐up interview at 1 month after successful resuscitation using the Cerebral Performance Category scale: category 1, good cerebral performance; category 2, moderate cerebral disability; category 3, severe cerebral disability; category 4, coma or vegetative state; and category 5, death/brain death.^17^, ^18^, ^19^ The data were integrated into the registry system on the Fire and Disaster Management Agency database server and subsequently had logical checks by the computer‐operated system. When the data form was not completed, the Fire and Disaster Management Agency contacted the responding EMS with instructions to complete the form.

### Outcome Measurements

The primary outcome was receiving layperson CPR. Secondary outcomes included 1‐month survival and favorable functional status at 1 month, defined as Cerebral Performance Category scale 1 or 2.^17^, ^18^, ^19^

### Statistical Analysis

First, we reported patient characteristics, stratified by sex. We presented continuous variables with both median and interquartile ranges and categorical variables with proportion. We performed Wilcoxon rank‐sum tests for continuous variables and chi‐squared tests for categorical variables to examine sex differences in patient characteristics.

Second, we reported sex differences in proportions receiving layperson CPR and stratified by type of layperson CPR: chest compression–only CPR and conventional CPR. We also stratified sex differences in proportions receiving layperson CPR by characteristics of witnesses: family versus nonfamily. Nonfamily included friends, colleagues, passersby, and others. EMS personnel who resuscitated patients recorded characteristics of witnesses. We performed chi‐squared tests to examine sex differences in proportions of receiving layperson CPR across types of layperson CPR.

Third, we fitted univariable and multivariable logistic regression models to predict sex differences in receiving layperson CPR. In the multivariable regression models we included age group (infants [0 year], children [1‐11 years], and adolescents [12‐17 years]), time of day of arrest (daytime/night),^21^ year,^14^ witnesses, and dispatcher CPR instruction^22^ as covariates. We included these covariates because these variables potentially affect provision of layperson CPR.^14^, ^21^, ^22^ Additionally, we fitted regression models with the same covariates and 2 interaction terms between patient sex and age groups and between patient sex and witnesses to evaluate effect of patient sex on receiving layperson CRP across age groups and witnesses. We reported ORs with 95% CIs. We stratified the regression models by age groups: infants (0 year), children (1‐11 years), and adolescents (12‐17 years).^23^ We also performed stratified analysis by witnesses (family or nonfamily) and repeatedly fitted univariable and multivariable regression models with the same covariates. We next employed multivariable logistic regression models to predict sex difference in secondary outcomes: 1‐month survival and favorable functional status at 1 month. These models adjusted for age, first documented rhythm, etiology, dispatcher CPR instruction, public automated external defibrillator shock delivery, layperson CPR, and EMS response time (interval from call to EMS arrival). We chose the covariates in the regression models a priori, based on their association with outcomes, biologic plausibility, and adequate ascertainment.^7^, ^8^, ^10^, ^21^, ^22^, ^23^, ^24^ All statistical analyses were performed using SPSS statistical package version 25.0J (IBM Corp, Armonk, NY). All tests were 2‐tailed, and *P*<0.05 was considered statistically significant.

## Results

**Figure 1:**
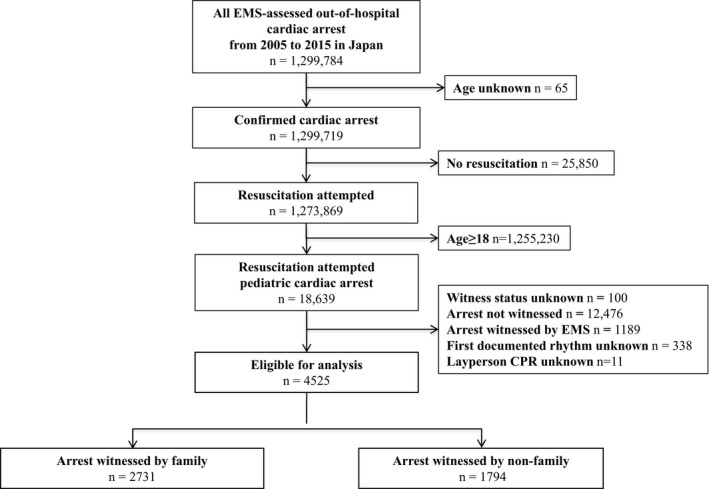
Study participant flowchart. CPR indicates cardiopulmonary resuscitation; EMS, emergency medical services.

A total of 1 299 784 OHCAs occurred during the study period, of whom 18 639 pediatric OHCA patients had resuscitation attempts by EMS personnel (Figure 1). After excluding those who met the exclusion criteria, 4525 patients with OHCA were eligible for our analyses.

Table 1 demonstrates patient characteristics by sex. Female patients were more likely to be younger, be witnessed by family, and to have nonshockable rhythm.

**Table 1:** Patient Characteristics By Sex

	Female (n=1669)	Male (n=2856)	*P* Values^a^
Age, y, median (IQR)	3 (0‐12)	7 (1‐15)	<0.001
Age category, n (%)			
Infants (0 y)	497 (29.8)	634 (22.2)	<0.001
Children (1‐11 y)	749 (44.9)	1107 (38.8)	
Adolescents (12‐17 y)	423 (25.3)	1115 (39.0)	
Characteristics of witnesses, n (%)			
Family	1144 (68.5)	1587 (55.6)	<0.001
Friends	76 (4.6)	317 (11.1)	
Colleagues	3 (0.2)	43 (1.5)	
Passersby	132 (7.9)	287 (10.0)	
Others	314 (18.8)	622 (21.8)	
Season, n (%)			
Spring	415 (24.9)	704 (24.6)	0.002
Summer	364 (21.8)	759 (26.6)	
Autumn	431 (25.8)	657 (23.0)	
Winter	459 (27.5)	736 (25.8)	
Time of day, n (%)			
Daytime (9:00 am to 4:59 pm)	691 (41.4)	1247 (43.7)	0.138
Night (5:00 pm to 8:59 am)	978 (58.6)	1609 (56.3)	
First documented rhythm, n (%)			
VF/pulseless VT	187 (11.2)	431 (15.1)	<0.001
PEA	514 (30.8)	772 (27.0)	
Asystole	968 (58.0)	1653 (57.9)	
Etiology, n (%)			
Cardiac etiology	611 (36.6)	956 (33.5)	0.032
Noncardiac etiology	1058 (63.4)	1900 (66.5)	
Dispatcher CPR instruction, n (%)	699 (42.3)	1104 (39.3)	0.050
Epinephrine, n (%)	49 (3.0)	121 (4.4)	0.094
Advanced airway management, n (%)	1317 (78.7)	2276 (79.7)	0.436
EMS resuscitation time, min, median (IQR)			
EMS response time (call to contact with patient)	8 (6‐10)	8 (6‐10)	0.087
Hospital arrival time (call to hospital arrival)	28 (22‐37)	28 (22‐37)	0.418

CPR indicates cardiopulmonary resuscitation; EMS, emergency medical services; IQR, interquartile range; PEA, pulseless electrical activity; VF, ventricular fibrillation; VT, ventricular tachycardia.

a Comparison between the 2 groups were evaluated with Wilcoxon rank‐sum test for continuous variables and chi‐squared test for categorical variables.

We reported unadjusted association between patient sex and receiving layperson CPR in Table 2. Female patients received layperson CPR in overall layperson‐witnessed OHCA more often than male patients (female 831/1669 [49.8%] versus male 1336/2856 [46.8%], *P*=0.05). Among those with arrest witnessed by family, female patients were significantly more likely to have received layperson CPR (female 600/1144 [52.4%] versus male 745/1587 [46.9%], *P*=0.005). Among those with arrest witnessed by nonfamily, there was no significant sex difference in receiving layperson CPR (female 231/525 [44.0%] versus male 591/1269 [46.6%], *P*=0.32). We did not observe significant sex difference in receiving layperson CPR in overall layperson‐witnessed OHCA between types of CPR: chest compression–only CPR (female 468/1669 [28.0%] versus male 746/2856 [26.1%], *P*=0.144) and conventional CPR (female 363/1669 [21.7%] versus male 590/2856 [20.7%], *P*=0.144).

**Table 2:** Difference in Receiving Layperson CPR by Patient Sex

	Overall Layperson‐Witnessed Arrest			Arrest Witnessed by Family			Arrest Witnessed by Nonfamily		
	Female (n=1669)	Male (n=2856)	*P* Values^a^	Female (n=1144)	Male (n=1587)	*P* Values^a^	Female (n=525)	Male (n=1269)	*P* Values^a^
Layperson CPR, n (%)	831 (49.8)	1336 (46.8)	0.050	600 (52.4)	745 (46.9)	0.005	231 (44.0)	591 (46.6)	0.320
Types of CPR									
Chest compression–only CPR	468 (28.0)	746 (26.1)	0.144	352 (30.8)	434 (27.3)	0.018	116 (22.1)	312 (24.6)	0.492
Conventional CPR	363 (21.7)	590 (20.7)		248 (21.7)	311 (19.6)		115 (21.9)	279 (22.0)	
Public‐access AED use	31 (1.9)	105 (3.7)	0.001	4 (0.3)	5 (0.3)	0.876	27 (5.1)	100 (7.9)	0.040

AED indicates automated external defibrillator; CPR, cardiopulmonary resuscitation.

a Comparison between the 2 groups was evaluated with chi‐squared test.

**Figure 2:**
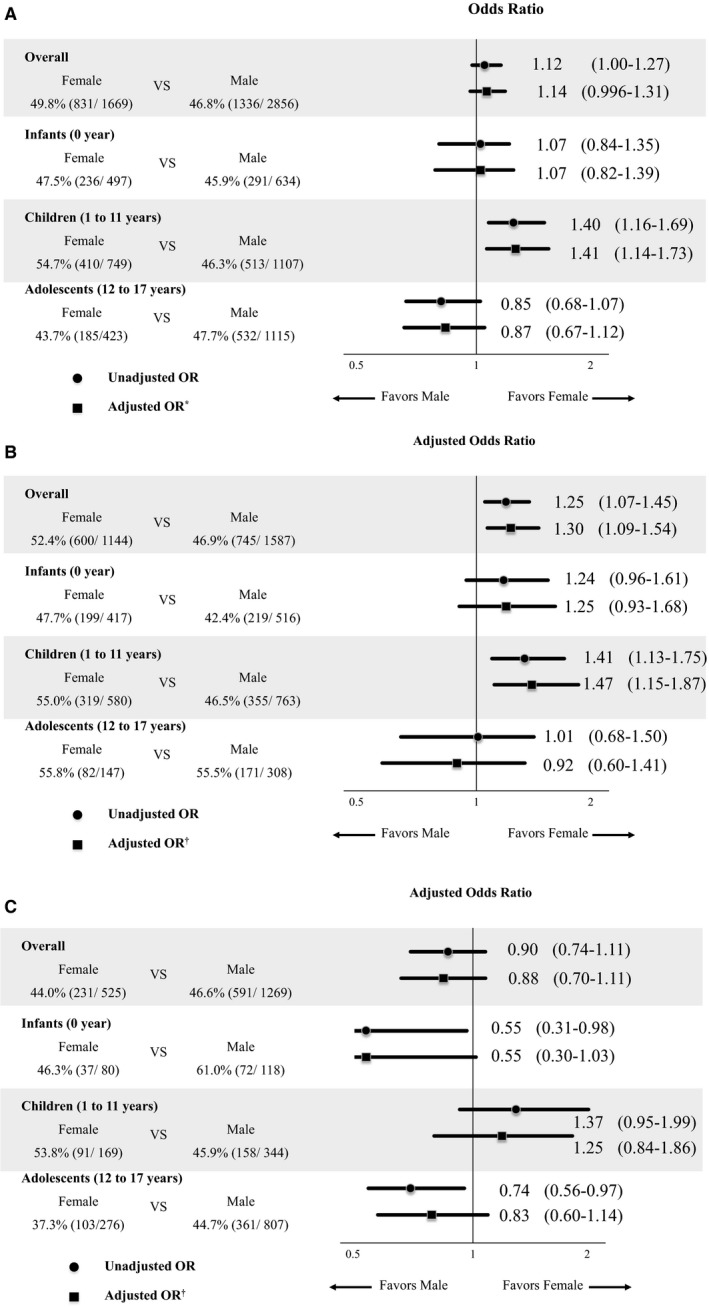
Association between receiving layperson cardiopulmonary resuscitation and sex of the patient, stratified by age group. Overall layperson‐witnessed arrest (**A**), arrest witnessed by family (**B**), and arrest witnessed by nonfamily (**C**). *Adjusted for age group, time of day of arrest, year, witnesses, and dispatcher CPR instruction. ^†^Adjusted for age group, time of day of arrest, year, and dispatcher CPR instruction. OR indicates odds ratio.

In multivariable analyses female patients were more likely to have received layperson CPR in arrest witnessed by family (adjusted OR=1.30; 95% CI 1.09‐1.54) (Figure 2B), whereas there were no significant sex differences in overall layperson‐witnessed OHCA (adjusted OR=1.14; 95% CI 0.996‐1.31) and in arrests witnessed by nonfamily (adjusted OR=0.88; 95% CI 0.70‐1.11) (Figure 2A and 2C). The interaction between patient sex and witnesses was significant (*P*‐value for interaction=0.038). After stratifying by age group in overall layperson‐witnessed arrest, female children (1‐11 years) received layperson CPR more often than male children (adjusted OR=1.41: 95% CI 1.14‐1.73) (Figure 2A). Male adolescents (12‐17 years) tended to receive layperson CPR more often than female adolescents without statistical significance (adjusted OR=0.87: 95% CI 0.67‐1.12) (Figure 2A). The interaction between patient sex and age group was not significant (*P*‐values for interaction=0.894). In arrest witnessed by nonfamily, male infants (0 year) and adolescents (12‐17 years) received layperson CPR more often than female infants and adolescents, although there was no statistical significance (Figure 2C).

We found that 18.9% (315/1669) of female and 17.8% (507/2856) of male patients had 1‐month survival (Table 3). After adjustment for covariates, there was no association between patient sex and 1‐month survival (adjusted OR for female patients=1.19; 95% CI 0.997‐1.42). We found that 7.2% (120/1669) of female and 8.1% (230/2856) of male patients had favorable functional outcomes. After adjustment, there was no association between patient sex and favorable functional status at 1 month (adjusted OR for female patients=1.29; 95% CI 0.98‐1.71). Among adolescents, female sex was associated with favorable survival and functional outcomes.

**Table 3:** Sex Differences in Secondary Outcomes After OHCA, Stratified by Age Group

	Overall Layperson‐Witnessed Arrest		Arrest Witnessed by Family		Arrest Witnessed by Nonfamily	
	Female (n=1669)	Male (n=2856)	Female (n=1144)	Male (n=1587)	Female (n=525)	Male (n=1269)
All age groups						
1‐mo survival, n (%)	315 (18.9)	507 (17.8)	215 (18.8)	303 (19.1)	100 (19.0)	204 (16.1)
Crude OR (95% CI)	1.08 (0.92‐1.26)	Reference	0.98 (0.81‐1.19)	Reference	1.23 (0.94‐1.60)	Reference
Adjusted OR (95% CI)^a^	1.19 (0.997‐1.42)	Reference	1.05 (0.85‐1.30)	Reference	1.53 (1.11‐2.12)	Reference
Favorable functional status at 1 mo (CPC 1 or 2), n (%)	120 (7.2)	230 (8.1)	60 (5.2)	102 (6.4)	60 (11.4)	128 (10.1)
Crude OR (95% CI)	0.89 (0.70‐1.11)	Reference	0.81 (0.58‐1.12)	Reference	1.15 (0.83‐1.59)	Reference
Adjusted OR (95% CI)^a^	1.29 (0.98‐1.71)	Reference	0.96 (0.67‐1.39)	Reference	2.02 (1.30‐3.14)	Reference
Infants (0 y)						
1‐mo survival, n (%)	93 (18.7)	104 (16.4)	82 (19.7)	89 (17.2)	11 (13.8)	15 (12.7)
Crude OR (95% CI)	1.17 (0.86‐1.60)	Reference	1.17 (0.84‐1.64)	Reference	1.10 (0.48‐2.53)	Reference
Adjusted OR (95% CI)^b^	1.17 (0.84‐1.63)	Reference	1.13 (0.80‐1.59)	Reference	0.85 (0.31‐2.36)	Reference
Favorable functional status at 1 mo (CPC 1 or 2), n (%)	18 (3.6)	32 (5.0)	15 (3.6)	28 (5.4)	3 (3.8)	4 (3.4)
Crude OR (95% CI)	0.71 (0.39‐1.28)	Reference	0.65 (0.34‐1.23)	Reference	1.11 (0.24‐5.10)	Reference
Adjusted OR (95% CI)^b^	0.79 (0.43‐1.47)	Reference	0.65 (0.34‐1.25)	Reference	1.71 (0.19‐15.15)	Reference
Children (1‐11 y)						
1‐mo survival, n (%)	119 (15.9)	176 (15.9)	94 (16.2)	132 (17.3)	25 (14.8)	44 (12.8)
Crude OR (95% CI)	1.00 (0.78‐1.29)	Reference	0.93 (0.69‐1.24)	Reference	1.18 (0.70‐2.01)	Reference
Adjusted OR (95% CI)^b^	1.02 (0.77‐1.34)	Reference	0.97 (0.71‐1.34)	Reference	1.04 (0.59‐1.86)	Reference
Favorable functional status at 1 mo (CPC 1 or 2), n (%)	37 (4.9)	44 (4.0)	29 (5.0)	30 (3.9)	8 (4.7)	14 (4.1)
Crude OR (95% CI)	1.26 (0.80‐1.96)	Reference	1.29 (0.76‐2.17)	Reference	1.17 (0.48‐2.85)	Reference
Adjusted OR (95% CI)^b^	1.36 (0.83‐2.24)	Reference	1.39 (0.78‐2.45)	Reference	0.78 (0.23‐2.66)	Reference
Adolescents (12‐17 y)						
1‐mo survival, n (%)	103 (24.3)	227 (20.4)	39 (26.5)	82 (26.6)	64 (23.2)	145 (18.0)
Crude OR (95% CI)	1.26 (0.97‐1.64)	Reference	1.00 (0.64‐1.55)	Reference	1.38 (0.99‐1.92)	Reference
Adjusted OR (95% CI)^b^	1.62 (1.16‐2.27)	Reference	1.08 (0.65‐1.81)	Reference	2.18 (1.37‐3.48)	Reference
Favorable functional status at 1 mo (CPC 1 or 2), n (%)	65 (15.4)	154 (13.8)	16 (11.0)	44 (14.3)	49 (17.8)	110 (13.6)
Crude OR (95% CI)	1.14 (0.83‐1.56)	Reference	0.74 (0.40‐1.36)	Reference	1.37 (0.95‐1.98)	Reference
Adjusted OR (95% CI)^b^	1.63 (1.07‐2.48)	Reference	0.81 (0.38‐1.72)	Reference	2.44 (1.43‐4.16)	Reference

AED indicates automated external defibrillator; CPC, cerebral performance category; CPR, cardiopulmonary resuscitation; EMS, emergency medical services; OHCA, out‐of‐hospital cardiac arrest; OR, odds ratio.

a Adjusted for age, public AED use, layperson CPR, dispatcher instruction, etiology of arrests, first documented rhythm, EMS response time.

b Adjusted for public AED use, layperson CPR, dispatcher instruction, etiology of arrests, first documented rhythm, EMS response time.

## Discussion

This observational study with a prospective, nationwide, population‐based registry including over 4500 pediatric patients with OHCA in Japan showed that female patients with layperson‐witnessed OHCA received layperson CPR more often than male patients, but there was no significant difference between patient sexes after adjustment for potential confounders. To our knowledge this is the first evaluation of sex differences in receiving layperson CPR among pediatric patients with OHCA, using a large data set.

An observational study using a nationwide OHCA registry in Denmark reported that female patients were less likely to receive layperson CPR than male patients among those who were ≥12 years old (median age; 72 years) and had EMS‐treated OHCA of cardiac cause (female 25.9% versus male 32.9%, *P*<0.001).^12^ Another observational study using the Resuscitation Outcomes Consortium, a multicenter OHCA registry in North America, showed that adult men received layperson CPR in public locations more than women (adjusted OR=1.27; 95% CI 1.05‐1.53) after adjustment for race, age, Resuscitation Outcomes Consortium sites, time of arrest, witness status, and EMS response time.^13^ In addition to the current knowledge from prior studies in adults with OHCA, we introduced important findings in regard to sex characteristics in receiving layperson CPR among a pediatric population.

In overall layperson‐witnessed arrest, male adolescents tended to receive layperson CPR more often than female adolescents, although the difference was not statistically significant. This implies that adolescents have similar findings to adults; ie, female adults with OHCA were less likely to receive layperson CPR than male adults.^12^, ^13^

We observed that male patients who were witnessed by family received layperson CPR less often than female patients (46.9% versus 52.4%, *P*=0.005), whereas among those who were witnessed by nonfamily, male patients tended to receive layperson CPR more often (46.6% versus 44.0%, *P*=0.32), a difference without statistical significance. Although this is an important finding, the underlying reasons for this difference are unclear, and further investigation is needed to understand the difference and increase provision of layperson CPR. Such work might include a qualitative approach, interviewing witness laypeople to extract the reason why CPR was or was not provided.

In overall layperson‐witnessed cardiac arrest, we did not find significant sex differences in 1‐month survival and favorable functional status at 1 month. An observational study using the Resuscitation Outcomes Consortium reported a rate of survival to hospital discharge of 10.2% without significant sex difference in 1738 pediatric patients with EMS‐treated OHCA (adjusted OR for male patients=0.73; 95% CI 0.44‐1.21).^23^ Similarly, another observational study using the Cardiac Arrest Registry to Enhance Survival, a national prospective voluntary registry of OHCA in the United States, demonstrated survival to hospital discharge rate of 8.2% without significant sex difference in survival to hospital discharge (adjusted OR for female patients=0.82; 95% CI 0.59‐1.14) and survival to hospital discharge with favorable neurologic outcome (adjusted OR for female patients=0.87; 95% CI 0.60‐1.27) among 1980 pediatric patients with EMS‐treated OHCA of presumed cardiac origin.^24^ Survival in our study was higher than that shown in prior reports, although direct comparison is not applicable because of differences in outcomes (ie, survival to hospital discharge in prior studies and 1‐month survival in our study) and study populations (ie, EMS‐treated OHCA in prior studies and layperson‐witnessed EMS‐treated OHCA in ours). Our results expand the findings in North America to another healthcare setting, using a larger sample size. A recent meta‐analysis of adult OHCA reported favorable outcomes in female patients.^25^ In the adult population biological sex difference such as female hormones was considered as 1 potential reason for favorable outcomes in female patients.^26^, ^27^ Favorable survival and functional outcomes in adolescent female patients in our study may reflect these findings from prior adult studies.

Our study has several clinical and public health implications. First, the observed sex differences in receiving layperson CPR should be a target of quality improvement efforts at the community level to increase the provision of layperson CPR for pediatric OHCA. Second, our results justify further efforts to identify underlying reasons for this observed sex difference in receiving layperson CPR. Such factors may include sex discordance in providing and receiving CPR (ie, sex difference in CPR providers and patients may affect provision of layperson CPR).

## Limitations

Our study has several limitations. First, there may have been unmeasured factors that confounded our results (eg, sex of layperson CPR providers). Second, our inference may not be fully generalizable to other healthcare settings, given the differences in patient characteristics and medical care systems. Third, in this data set, the sex of layperson CPR providers was not available, and we were unable to assess the effect of sex discordance and concordance between CPR providers and patients on receiving layperson CPR. Last, as with all epidemiological studies, data integrity, validity, and ascertainment bias are potential limitations. The use of uniform data collection based on the Utstein‐style guidelines for reporting cardiac arrest, large sample size, and a population‐based design to cover all OHCA in Japan were intended to minimize these potential biases.

## Conclusions

In this analysis of a prospective, nationwide, population‐based registry system of OHCA in Japan, receiving layperson CPR tended to be different between patient sexes, although there was no significant sex difference after adjustment for covariates. Witnesses impacted sex difference in receiving layperson CPR. Public health efforts to improve the provision of layperson CPR in pediatric patients should increase interventions for both sexes.

## Disclosures

None.
